# Molecular docking data of E3 ubiquitin-protein ligase WWP1 with compounds from a medicinal plant Justicia adhatoda L

**DOI:** 10.6026/97320630017162

**Published:** 2021-01-31

**Authors:** Jayaraman Selvaraj, Umapathy Vidhya Rekha, Shazia Fathima JH, Venkatacalam Sivabalan, Rajagopal Ponnulakshmi, Veeraraghavan Vishnu Priya, Malathi Kullappan, Radhika nalinakumari Sreekandan, Surapaneni Krishna Mohan

**Affiliations:** 1 Department of Biochemistry, Saveetha Dental College and Hospitals, Saveetha Institute of Medical and Technical Sciences, Saveetha University, Chennai - 600 077, India; 2 Department of Public Health Dentistry, Sree Balaji Dental College and Hospital, Pallikaranai,Chennai-600 100, India; 3 Department of Oral and Maxillofacial Pathology, Ragas Dental College and Hospitals, Chennai, India; 4 Department of Biochemistry, KSR Institute of Dental Sciences and Research, Thiruchengodu-637215, India; 5 Central Research Laboratory,Meenakshi Academy of Higher Education and Research (Deemed to be University), Chennai-600 078, India; 6 Department of Research,Panimalar Medical College Hospital & Research Institute, Varadharajapuram, Poonamallee, Chennai - 600 123, India; 7 Department of Clinical Skills & Simulation, Panimalar Medical College Hospital & Research Institute, Varadharajapuram, Poonamallee, Chennai - 600 123, India; 8 Department of Biochemistry and Department of Clinical Skills & Simulation, Department of Research, Panimalar Medical College Hospital & Research Institute, Varadharajapuram, Poonamallee, Chennai - 600 123, India

**Keywords:** Oral cancer, WWP1, Justicia adhatoda L, molecular docking

## Abstract

It is known that E3 ubiquitin-protein ligase WWP1 is linked to oral cancer. Therefore, it is of interest to document molecular docking data of E3 ubiquitin-protein ligase WWP1 with compounds ((Stigmasterol, Pyrazinamide, Vasicinone and Ethambutol)) from a medicinal plant Justicia adhatoda L for further consideration.

## Background:

Oral cancer is one of the most common malignancies [1-5]. It is known that E3 ubiquitin-protein ligase WWP1 is linked to oral cancer [6-12]. Therefore, it is of interest to document molecular docking data of E3 ubiquitin-protein ligase WWP1 with compounds ((Stigmasterol, Pyrazinamide, Vasicinone and Ethambutol)) from a medicinal plant Justicia adhatoda L for further consideration.

## Materials and Methods:

### Preparation of the protein structure:

The protein structure of WWP1 was downloaded from the Protein Data Bank at 2.1Å resolution (PDB: ID 1ND7) for this analysis.

### Ligand Preparation:

The compounds from Justicia adhatoda were downloaded from the PubChem database (Table 1 - see PDF) in (.sdf) format and converted to (.pdb) format using the online Smiles.

### Molecular Docking:

Molecular docking study was completed using AutoDock vina in The Python Prescription (PyRx) 0.8 virtual screening tool [13]. The grid points in the X, Y and Z-axes are set. The grid core was positioned in the pocket core of the binding site. Protein and ligands were translated to pdbqt formats. Default docking algorithms are used using standard docking protocol. Data is then ranked in the order of rising docking energies. The lowest binding energy of each cluster was considered further [14].

## Results and Discussion:

**Figure 1:**
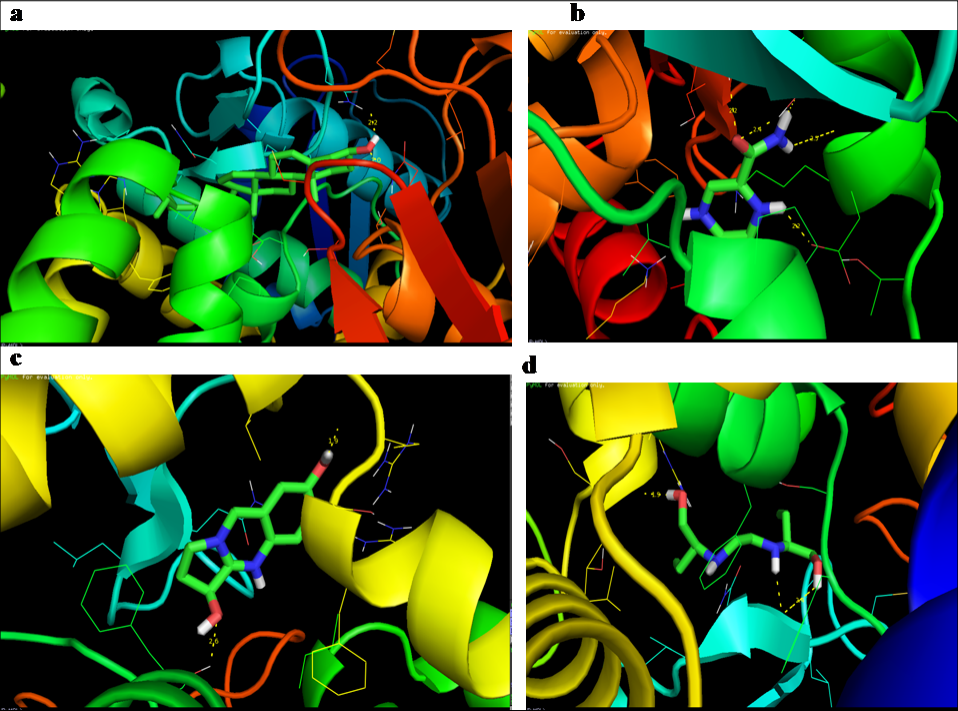
Molecular docking interaction of WWP1 with (a) Stigmasterol; (b) Pyrazinamide; (c) Vasicinone and (d) Ethambutol

Oral cancer is one of the most common malignancies [1-5]. It is known that E3 ubiquitin-protein ligase WWP1 is linked to oral cancer [6-12]. Therefore, it is of interest to document molecular docking data of E3 ubiquitin-protein ligase WWP1 with compounds from a medicinal plant Justicia adhatoda L for further consideration. Molecular docking analysis of 12 compounds (Table 1 - see PDF) from Justicia adhatoda E3 ubiquitin-protein ligase WWP1 was completed (Table 2 - see PDF). Stigmasterol (-10.21kcal/mol), Pyrazinamide (-8.6 kcal/mol), Vasicinone (-8.2 kcal/mol) and Ethambutol (-7.6 kcal/mol) showed good binding with the WWP1 gene protein target. The interaction of compounds and the target protein was visualized using PyMOL as shown in Figure 1. The amino acids residues MET-865, ASN-892, LYS-694, ASP-695, SER-698, THR-889, SER-679, ARG-767, LEU-641 and PHE-765 were involved in the interaction between the WWP1 and compounds through H-bond formation. Thus, we document molecular docking data of E3 ubiquitin-protein ligase WWP1 with compounds ((Stigmasterol, Pyrazinamide, Vasicinone and Ethambutol)) from a medicinal plant Justicia adhatoda L for further consideration in the context of oral cancer.

## Conclusion:

We document molecular docking data of E3 ubiquitin-protein ligase WWP1 with compounds ((Stigmasterol, Pyrazinamide, Vasicinone and Ethambutol)) from a medicinal plant Justicia adhatoda L for further consideration in the context of oral cancer.
